# Therapeutic potential of human umbilical cord mesenchymal stem cell-derived exosomes in myocardial infarction: from molecular mechanisms to clinical translation-an update

**DOI:** 10.3389/fphar.2025.1667140

**Published:** 2025-09-10

**Authors:** Nianhui Ding, Zaiyong Zheng, Chunxiang Zhang

**Affiliations:** ^1^ School of Pharmacy, Southwest Medical University, Luzhou, Sichuan, China; ^2^ Anesthesiology and Critical Care Medicine Key Laboratory of Luzhou, The Affiliated Hospital of Southwest Medical University, Luzhou, Sichuan, China; ^3^ Department of Cardiology, Key Laboratory of Medical Electrophysiology, Basic Medicine Research Innovation Center for Cardiometabolic Diseases, The Affiliated Hospital of Southwest Medical University, Southwest Medical University, Luzhou, China

**Keywords:** exosomes, myocardial infarction, cardiac regeneration, tissue engineering, therapeutic angiogenesis, human umbilical cord mesenchymal stem cells

## Abstract

Myocardial infarction (MI) remains a leading cause of cardiovascular mortality despite advances in reperfusion strategies, necessitating innovative therapeutic approaches. Human umbilical cord mesenchymal stem cell-derived exosomes (HUCMSCs-Exos) have emerged as promising next-generation therapeutics, offering superior advantages including enhanced stability, reduced immunogenicity, and ability to cross biological barriers compared to cellular therapies. These naturally occurring nanovesicles exert comprehensive cardioprotective effects through multifaceted mechanisms encompassing anti-apoptotic signaling, angiogenesis promotion, immunomodulation, anti-fibrotic activity, oxidative stress reduction, and cardiac regeneration enhancement. The therapeutic arsenal includes diverse molecular cargo such as microRNAs (miR-29b, miR-133a-3p, miR-24-3p), long non-coding RNAs, circular RNAs, and bioactive proteins that synergistically target key pathophysiological processes in MI. Advanced engineering approaches, including genetic modification, surface functionalization, and biomaterial integration, have further enhanced therapeutic efficacy through targeted delivery and sustained release systems. While preclinical studies demonstrate significant cardioprotective effects, clinical translation faces challenges in standardization, manufacturing scalability, and regulatory approval. The convergence of innovative engineering strategies, personalized medicine approaches, and emerging technologies positions HUCMSCs-Exos as promising therapeutic approach that could fundamentally alter MI treatment paradigms and improve global cardiovascular health outcomes.

## 1 Introduction

Myocardial infarction (MI) remains one of the leading causes of morbidity and mortality worldwide, representing a major public health challenge despite significant advances in cardiovascular medicine over the past decades (Ong et al., 2024). The pathophysiology of MI involves a complex cascade of events, typically initiated by atherosclerotic plaque rupture or erosion, leading to coronary thrombosis and subsequent myocardial tissue necrosis (Reed et al., 2017). While contemporary reperfusion strategies, including percutaneous coronary intervention (PCI) and thrombolytic therapy, have dramatically improved survival rates and reduced the size of MI (Reddy et al., 2024), substantial challenges persist in clinical practice.

Despite optimal guideline-directed medical therapy and timely reperfusion, patients who develop cardiogenic shock following MI continue to face mortality rates exceeding 40% (Abu Ghosh et al., 2023). The pathogenesis of MI is fundamentally rooted in atherosclerosis, a chronic inflammatory process affecting the arterial wall. This process involves complex interactions between endothelial dysfunction, lipid accumulation, inflammatory cell infiltration, and thrombosis (Dong et al., 2024). The vulnerable plaque, characterized as a thin-cap fibroatheroma with a lipid-rich necrotic core, is prone to rupture and subsequent thrombotic events (He et al., 2023). Understanding these mechanisms has led to improved preventive strategies and acute management protocols, yet the regenerative capacity of the adult myocardium remains limited, necessitating innovative therapeutic approaches.

In recent years, cell-based therapies, particularly those utilizing mesenchymal stem cells (MSCs), have emerged as promising strategies for cardiovascular regeneration (Lee et al., 2021). MSCs possess remarkable therapeutic potential due to their multi-lineage differentiation capacity, immunomodulatory properties, and paracrine effects. However, growing evidence suggests that the therapeutic benefits of MSCs are primarily mediated through their paracrine mechanisms rather than direct cellular engraftment and differentiation (Zhidu et al., 2024). This paradigm shift has focused scientific attention toward extracellular vesicles, particularly exosomes, as the key mediators of MSC therapeutic effects. Compared to MSCs, exosomes retain the therapeutic functions of their parent cells while offering distinct advantages including enhanced long-term stability, efficient internalization into recipient cells, minimal immunogenic rejection, and convenient administration routes (Huang P. et al., 2020). Furthermore, both fresh and cryopreserved exosomes demonstrate comparable efficacy in promoting angiogenesis and suppressing lymphocyte proliferation *in vitro*, whereas depletion of exosomes from MSC-conditioned media substantially diminishes their capacity to ameliorate MI, further substantiating the pivotal role of exosomes as primary mediators of MSC-induced cardioprotection (Teng et al., 2015).

**FIGURE 1:**
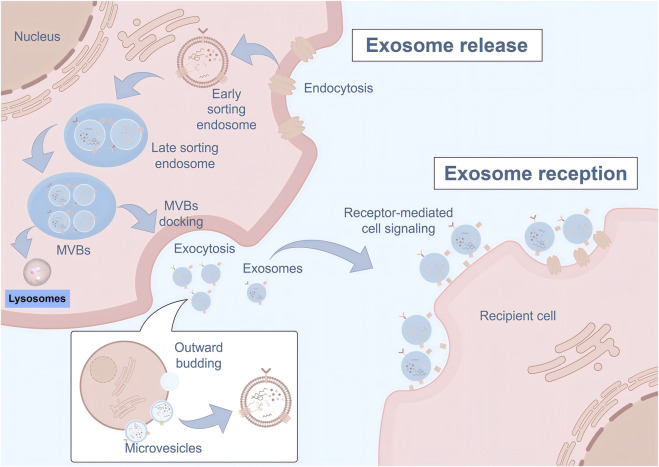
Biogenesis and intercellular communication pathways of exosomes. The schematic diagram illustrates the complete process of exosome biogenesis, release, and uptake. Exosome formation begins with the invagination of the plasma membrane to form early sorting endosomes, which subsequently mature into late sorting endosomes. These structures then develop into multivesicular bodies (MVBs) containing intraluminal vesicles through inward budding of the endosomal membrane. MVBs can either fuse with lysosomes for degradation or dock with the plasma membrane to release exosomes via exocytosis. Additionally, microvesicles are generated through direct outward budding of the plasma membrane. Released exosomes mediate intercellular communication through receptor-mediated cell signaling pathways, allowing for the transfer of bioactive cargo including proteins, nucleic acids, and lipids to recipient cells, thereby modulating target cell function and behavior.

Exosomes are naturally occurring, membrane-bound nanovesicles (40–200 nm) that serve as crucial mediators of intercellular communication. These vesicles carry diverse cargo including proteins, lipids, mRNAs, microRNAs (miRNAs), and other bioactive molecules that can modulate target cell behavior and function (Krylova and Feng, 2023). Compared to their parent cells, exosomes offer several distinct advantages as therapeutic agents, including lower immunogenicity, enhanced stability, ability to cross biological barriers, and reduced risk of malignant transformation (Zhang et al., 2025). The biogenesis and absorption of exosomes are summarized in Figure 1.

Among various sources of MSCs, human umbilical cord mesenchymal stem cells (HUCMSCs) have garnered particular attention due to their unique advantages. HUCMSCs can be obtained through minimally invasive procedures without ethical concerns, demonstrate superior self-renewal capacity and proliferation potential compared to other MSC sources, and exhibit lower immunogenicity (Li et al., 2015; Bárcia et al., 2015). These characteristics make HUCMSCs an ideal source for large-scale exosome production for clinical applications.

Recent preclinical studies have demonstrated that HUCMSCs-derived exosomes (HUCMSCs-Exos) exert cardioprotective effects through multiple mechanisms, including promotion of angiogenesis, reduction of cardiomyocyte apoptosis, modulation of inflammatory responses, and attenuation of adverse cardiac remodeling (Liu et al., 2023). The therapeutic potential of these exosomes extends beyond the cardiovascular system, with demonstrated efficacy in various disease models including pulmonary diseases, where they have shown remarkable success in treating conditions such as bronchopulmonary dysplasia through mechanisms involving immune regulation, anti-inflammatory responses, and tissue regeneration (Cheng et al., 2024). These findings provide a solid theoretical foundation for advancing the application of HUCMSCs-Exos in MI treatment.

This comprehensive review aims to synthesize current knowledge regarding the relationship between HUCMSCs-Exos and MI, exploring their potential mechanisms of action, therapeutic potential, and challenges facing clinical translation. Through comprehensive analysis of this rapidly evolving field, we seek to facilitate the translation of HUCMSCs-Exos from promising laboratory discoveries into effective clinical interventions for patients with MI.

## 2 Pathophysiology of MI: from molecular mechanisms to clinical manifestations

### 2.1 Atherosclerosis and plaque vulnerability

Atherosclerosis, the primary pathological substrate for MI, involves chronic arterial inflammation initiated by endothelial dysfunction and lipid accumulation (Aispur et al., 2025). Vulnerable plaques characterized by thin fibrous caps and large necrotic cores are prone to rupture through matrix metalloproteinase-mediated degradation, with inflammatory cytokines from activated macrophages driving plaque instability (Korjian et al., 2023; Wan et al., 2023).

### 2.2 Thrombosis and coronary occlusion

Plaque rupture or erosion exposes thrombogenic material, triggering platelet activation and coagulation cascade through tissue factor exposure (Abubakar et al., 2023). While rupture accounts for 70% of acute coronary syndromes, plaque erosion represents an important alternative mechanism, particularly in younger patients and women (Arbustini et al., 1999).

### 2.3 Myocardial ischemia-reperfusion injury

Coronary occlusion rapidly depletes myocardial high-energy phosphate stores, shifting metabolism from aerobic to anaerobic pathways with consequent lactate accumulation and acidosis (Wang J. et al., 2024). Paradoxically, reperfusion can exacerbate injury through reactive oxygen species generation, calcium overload, and mitochondrial permeability transition pore opening (Algoet et al., 2023). The no-reflow phenomenon, affecting up to 30% of primary PCI patients, results from microvascular dysfunction despite successful epicardial recanalization (Wang R. et al., 2024).

### 2.4 Inflammatory cascade and immune responses

MI triggers biphasic inflammation essential for both injury and healing. Initial neutrophil infiltration and damage-associated molecular pattern recognition through Toll-like receptors amplify tissue damage (Arslan et al., 2011). The NLR family pyrin domain containing 3 (NLRP3) inflammasome mediates sterile inflammation through interleukin-1β (IL-1β) and IL-18 release, representing a key therapeutic target for HUCMSCs-Exos (Toldo and Abbate, 2024). Successful healing requires timely transition from pro-inflammatory M1 to reparative M2 macrophages, a process modulated by IL-10 and transforming growth factor beta (TGF-β) that HUCMSCs-Exos can enhance (Jia et al., 2022).

### 2.5 Cardiac remodeling and heart failure progression

Post-MI remodeling encompasses both infarct-specific and global ventricular changes. Early adaptive responses include compensatory hypertrophy and infarct expansion, while maladaptive remodeling features progressive dilatation, excessive fibrosis, and neurohormonal activation (Ai et al., 2025). The extracellular matrix undergoes time-dependent changes with initial degradation followed by collagen deposition, processes directly targeted by HUCMSCs-Exos therapeutic mechanisms (Lindsey et al., 2016; Sapna et al., 2023).

## 3 Biology and characteristics of human umbilical cord mesenchymal stem cell-derived exosomes

### 3.1 Biogenesis and secretion pathways

HUCMSCs-derived exosomes are generated through endosomal maturation involving endosomal sorting complex required for transport-dependent and independent pathways. The process begins with plasma membrane invagination forming early endosomes, which mature into multivesicular bodies containing intraluminal vesicles (Cheng et al., 2024). Exosome release occurs through multivesicular body fusion with the plasma membrane, regulated by Rab GTPases and influenced by cellular stress and cytokine stimulation (Guo et al., 2024).

### 3.2 Structural composition and cargo content

#### 3.2.1 Proteins and enzymes

HUCMSCs-Exos contain more than 400 proteins, including tetraspanins (CD63, CD81, CD9), heat shock proteins (HSP70, HSP90), and antioxidant enzymes (catalase, superoxide dismutase) (Cheng et al., 2024; Zhang et al., 2016; Kodali et al., 2024). These proteins contribute to membrane fusion, protein stabilization, and cytoprotective functions essential for therapeutic efficacy.

#### 3.2.2 Nucleic acids and cytokines

The nucleic acid cargo includes >200 miRNAs with cardioprotective properties (miR-21, miR-146a, miR-423) (Cheng et al., 2024; Bi et al., 2022). Furthermore, HUCMSCs-Exos contain abundant cytokines, including granulocyte-macrophage colony-stimulating factor, IL-15, IL-6, IL-8, tumor necrosis factor-α, IL-1β, IL-2, and IL-10, with particularly high concentrations of IL-6 and IL-8 (Zhang et al., 2016). These molecules modulate recipient cell gene expression, promoting survival, angiogenesis, and anti-inflammatory responses.

#### 3.2.3 Lipids and metabolites

HUCMSCs-Exos are enriched in cholesterol, sphingomyelin, and bioactive lipids that enhance membrane stability and anti-inflammatory properties. The metabolite cargo includes amino acids and energy metabolites that influence recipient cell metabolism (Cheng et al., 2024; Cortes-Galvez et al., 2023).

### 3.3 Isolation and purification techniques

Ultracentrifugation remains the most commonly employed technique for initial HUCMSCs-Exos isolation. However, it is important to note that exosomes isolated through ultracentrifugation may be contaminated with non-vesicular macromolecules, which can significantly impact omic analyses and functional assessments of exosomes. Nevertheless, studies have reported that chromatography-based isolation methods result in lower contamination levels of non-vesicular proteins and macromolecular structures compared to ultracentrifugation, providing research directions for further optimization of HUCMSCs-Exos extraction protocols (Gardiner et al., 2016). Furthermore, advanced techniques including asymmetric flow field-flow fractionation offer scalable alternatives for clinical production with enhanced purity and reproducibility (Yang et al., 2019).

### 3.4 Characterization and quality control

Comprehensive characterization encompasses physical parameters (size distribution by nanoparticle tracking analysis/dynamic light scattering, morphology by transmission electron microscopy), biochemical markers (CD63, CD81, CD9, Alix), and functional assays assessing biological activity (Welsh et al., 2024). Standardized protocols ensure consistency and therapeutic potential across production batches.

### 3.5 Advantages over other MSC sources

Comparative analysis of exosomes from different MSC sources reveals distinct advantages of HUCMSCs-Exos for cardiac applications. While bone marrow-derived MSC exosomes demonstrate therapeutic efficacy, they exhibit age-related functional decline and require invasive harvesting procedures that limit clinical scalability (Li et al., 2015; Bárcia et al., 2015). Cardiac progenitor cell-derived exosomes offer tissue-specific advantages but face critical limitations in source availability and expansion capacity (Barile et al., 2014). HUCMSCs demonstrate superior proliferative capacity with population doubling times of 30–40 h versus 50–70 h for bone marrow MSCs, and exhibit lower immunogenicity with minimal HLA-DR expression (Li et al., 2015; Bárcia et al., 2015; Jabbehdari et al., 2020). Recent proteomic analyses demonstrate that HUCMSCs-Exos contain higher concentrations of pro-angiogenic factors including VEGF and angiopoietin-1 compared to other MSC sources (Zhang et al., 2016; Kodali et al., 2024; Bi et al., 2022). These findings establish a robust theoretical foundation for advancing HUCMSCs-Exos applications across various disease states. Subsequently, we will comprehensively examine the cardioprotective effects of HUCMSCs-Exos in MI and their underlying mechanisms based on existing research findings, with the aim of providing theoretical foundation and support for further advancing the clinical translation of HUCMSCs-Exos.

## 4 Therapeutic mechanisms of HUCMSCs-Exos in MI

**FIGURE 2:**
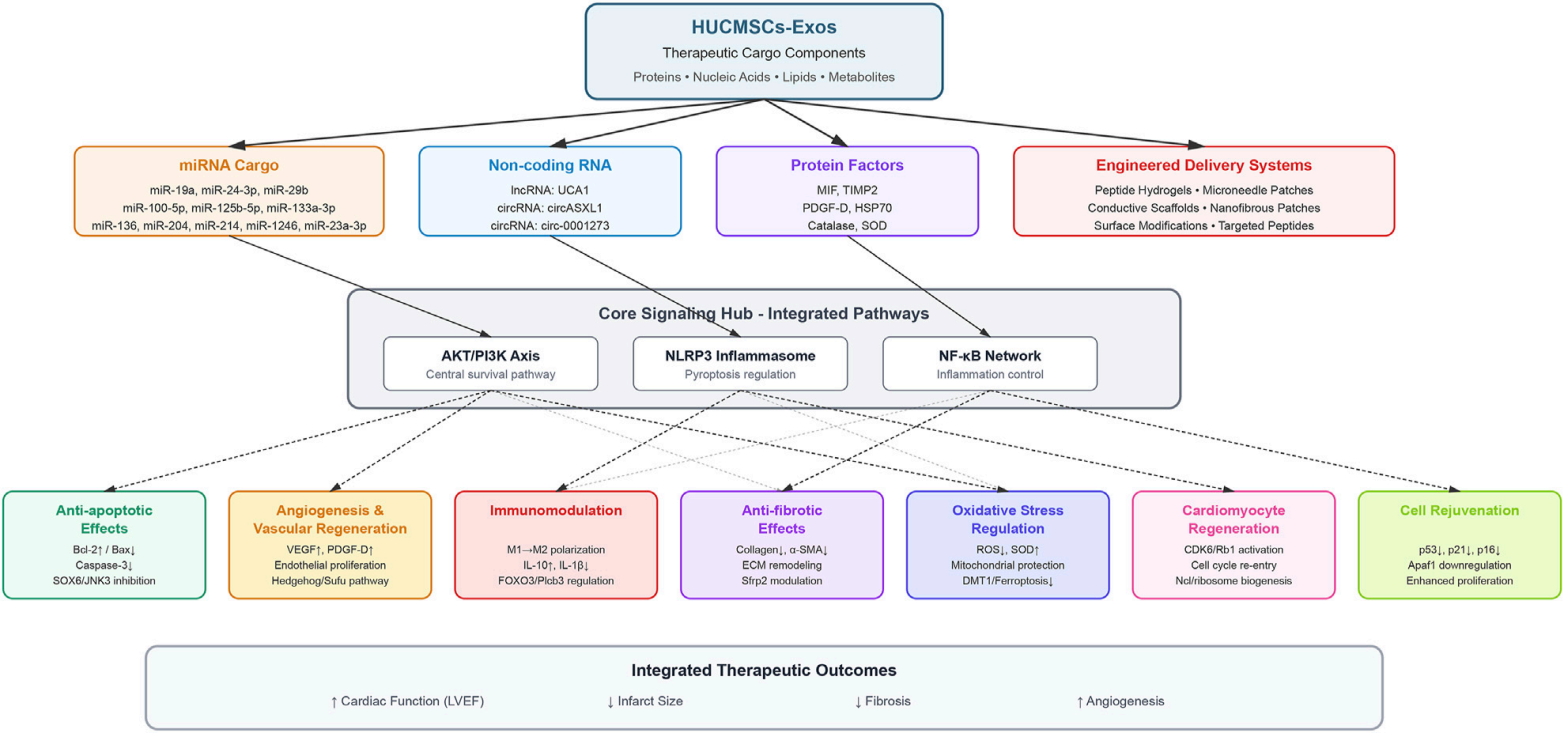
Integrated signaling network and therapeutic mechanisms of HUCMSCs-Exos in MI. This hierarchical cascade diagram illustrates the systematic flow of therapeutic signals from HUCMSCs-Exos to clinical outcomes. The top layer depicts HUCMSCs-Exos as the central therapeutic platform containing diverse bioactive cargo. The second layer categorizes four major cargo components: (1) miRNA cargo; (2) non-coding RNAs; (3) protein factors; and (4) engineered delivery systems. The third layer represents the core signaling hub integrating three major shared pathways—AKT/PI3K axis (central survival pathway), NLRP3 inflammasome (pyroptosis regulation), and NF-κB network (inflammation control)—thereby eliminating pathway redundancy. The fourth layer displays seven distinct therapeutic effects mediated by the integrated signaling networks: anti-apoptotic effects, angiogenesis and vascular regeneration, immunomodulation, anti-fibrotic effects, oxidative stress regulation, cardiomyocyte regeneration, and cell rejuvenation. Solid arrows indicate direct activation and dashed arrows represent indirect regulation. The bottom panel summarizes the integrated therapeutic outcomes including improved cardiac function (LVEF), reduced infarct size, decreased fibrosis, and enhanced angiogenesis. Abbreviations: AKT, protein kinase B; Apaf1, apoptotic peptidase activating factor 1; Bax, Bcl-2-associated X protein; Bcl-2, B-cell lymphoma 2; CDK6, cyclin-dependent kinase 6; circRNA, circular RNA; DMT1, divalent metal transporter 1; ECM, extracellular matrix; FOXO3, forkhead box O3; HSP70, heat shock protein 70; IL, interleukin; JNK3, c-Jun N-terminal kinase 3; lncRNA, long non-coding RNA; LVEF, left ventricular ejection fraction; MIF, macrophage migration inhibitory factor; miR, microRNA; Ncl, nucleolin; NF-κB, nuclear factor kappa B; NLRP3, NLR family pyrin domain containing 3; PDGF-D, platelet-derived growth factor D; PI3K, phosphoinositide 3-kinase; Plcb3, phospholipase C beta 3; Rb1, retinoblastoma protein 1; ROS, reactive oxygen species; Sfrp2, secreted frizzled-related protein 2; SOD, superoxide dismutase; SOX6, SRY-box transcription factor 6; Sufu, suppressor of fused; TIMP2, tissue inhibitor of metalloproteinases 2; UCA1, urothelial cancer associated 1; VEGF, vascular endothelial growth factor; α-SMA, alpha-smooth muscle actin.

The therapeutic efficacy of HUCMSCs-Exos in MI is mediated through multiple interconnected mechanisms that target the key pathophysiological processes underlying cardiac injury and repair. Recent advances have revealed the multifaceted nature of exosome-mediated cardioprotection, which encompasses cellular survival, tissue regeneration, and functional restoration. Notably, multiple therapeutic mechanisms converge on key signaling nodes, particularly the phosphoinositide 3-kinase/protein kinase B (AKT/PKB) pathway, which emerges as a central hub mediating anti-apoptotic, pro-angiogenic, and anti-fibrotic effects of HUCMSCs-Exos. Rather than representing redundancy, this convergence highlights the integrated nature of exosome-mediated cardioprotection, where single molecular cargo can trigger cascading effects across multiple pathways. Figure 2 illustrates the mechanism of action of HUCMSCs-Exos in preclinical models of MI.

### 4.1 Anti-apoptotic and cytoprotective effects

Cardiomyocyte apoptosis represents a critical pathological feature of MI, and HUCMSCs-Exos demonstrate remarkable anti-apoptotic properties through diverse molecular pathways. Zhao et al. showed that HUCMSCs-Exos significantly reduced cardiomyocyte apoptosis and improved cardiac systolic function, with effects potentially mediated through regulation of B cell lymphoma-2 (Bcl-2) family proteins (Zhao et al., 2015). Moreover, the cytoprotective mechanisms involve multiple miRNA-mediated pathways, including miR-19a targeting Sry-related high-mobility group box6 to activate AKT/PKB signaling while inhibiting c-Jun N-terminal kinase 3/caspase-3 activation (Huang L. et al., 2020).

Furthermore, HUCMSCs-Exos enriched in miR-125b-5p promote myocardial repair by upregulating Smad7 expression, effectively countering the downregulation observed in ischemic conditions (Wang et al., 2018). The long non-coding RNA UCA1 delivered by HUCMSCs-Exos provides additional cytoprotective effects by competitively binding miR-143, thereby preventing Bcl-2 degradation and maintaining cellular survival pathways (Diao and Zhang, 2021). These findings collectively demonstrate the comprehensive anti-apoptotic mechanisms employed by HUCMSCs-Exos to preserve cardiomyocyte viability in ischemic conditions.

### 4.2 Angiogenesis and vascular regeneration

Neovascularization is essential for cardiac repair following MI, and HUCMSCs-Exos exhibit potent pro-angiogenic properties through multiple complementary mechanisms. Genetic modification of HUCMSCs to overexpress macrophage migration inhibitory factor (MIF) significantly enhances the angiogenic potential of derived exosomes by upregulating miR-133a-3p, which subsequently activates the AKT signaling pathway in endothelial cells, thereby promoting angiogenesis (Zhu et al., 2021). Similarly, hypoxia-preconditioned MSCs produce exosomes enriched in miR-214, which promotes angiogenesis through the miR-214/Sufu pathway by activating hedgehog signaling in human umbilical vein endothelial cells. Concurrently, significant increases in capillary density were observed in MI rat models, suggesting that hypoxia-induced vesicle modification may represent a viable approach for restoring cardiac function and promoting angiogenesis following MI (Shao et al., 2023).

The angiogenic effects are further amplified through exosomal miR-1246, which potentiates myocardial angiogenesis in chronic heart failure by targeting serine protease 23 and inhibiting Snail/α-smooth muscle actin signaling (Wang et al., 2021). AKT-modified HUCMSCs produce exosomes with enhanced angiogenic capacity through upregulation of platelet-derived growth factor D (PDGF-D), demonstrating superior effects on endothelial cell proliferation, migration, and tube formation (Ma et al., 2017). These diverse pro-angiogenic mechanisms highlight the multifaceted approach employed by HUCMSCs-Exos to restore vascular networks in infarcted myocardium. The diverse repertoire of miRNAs carried within these exosomes represents a crucial mechanistic pathway through which HUCMSCs-Exos exert their pro-angiogenic effects.

### 4.3 Immunomodulation and inflammatory response regulation

The inflammatory response following MI requires precise modulation to prevent excessive tissue damage while promoting appropriate healing responses. HUCMSCs-Exos demonstrate sophisticated immunomodulatory capabilities, particularly through regulation of macrophage polarization. Exosomal miR-24-3p promotes M2 macrophage polarization by targeting phospholipase C-beta3 and suppressing nuclear factor-kappaB pathway activation, thereby reducing excessive inflammation and promoting tissue repair (Zhu et al., 2022). This effect is complemented by miR-204 carried by HUCMSCs-Exos, which similarly promotes M2 polarization while inhibiting pro-inflammatory M1 activation (Yuan et al., 2022).

The anti-inflammatory effects extend to the regulation of pyroptosis, a form of programmed cell death characterized by intense inflammatory responses. HUCMSCs-Exos modulate the NLRP3 inflammasome/caspase-1 pathway to suppress pyroptosis in cardiac microvascular endothelial cells exposed to hypoxia/reoxygenation injury (Diao et al., 2024). Additionally, exosomal miR-100-5p protects cardiomyocytes against hypoxia/reoxygenation-induced pyroptosis by targeting forkhead box O3 and inhibiting NLRP3 inflammasome activation (Liang et al., 2021).

These mechanisms collectively demonstrate the sophisticated anti-inflammatory arsenal of HUCMSCs-Exos, particularly pyroptosis associated with the NLRP3 inflammasome.

### 4.4 Anti-fibrotic effects and cardiac remodeling prevention

Cardiac fibrosis represents a major contributor to heart failure progression following MI, and HUCMSCs-Exos exhibit anti-fibrotic properties. Exosomal miR-29b demonstrates potent anti-fibrotic activity by downregulating fibrosis-related proteins and reducing excessive collagen deposition (Yuan et al., 2023). The anti-fibrotic effects are enhanced through the promotion of fibroblast-to-myofibroblast differentiation in inflammatory environments, which paradoxically provides cardioprotective benefits by shifting from pro-inflammatory to anti-inflammatory phenotypes (Shi et al., 2019).

Tissue inhibitor of metalloproteinases 2 (TIMP2)-engineered HUCMSCs produce exosomes with enhanced anti-remodeling properties through the AKT/secreted frizzled-related protein 2 (Sfrp2) pathway, effectively restricting extracellular matrix remodeling and reducing collagen deposition (Ni et al., 2019). These exosomes demonstrate enhanced therapeutic potential in preventing adverse cardiac remodeling while promoting beneficial tissue reorganization. Long-term studies demonstrate that HUCMSCs-Exos can reverse ventricular remodeling and improve cardiac function through sustained anti-fibrotic effects. These findings further extend both the duration and scope of HUCMSCs-Exos therapeutic applications (Wang et al., 2025).

### 4.5 Regulation of oxidative stress and cellular metabolism

Oxidative stress plays a central role in myocardial injury progression, and HUCMSCs-Exos demonstrate significant antioxidant properties. These exosomes contain antioxidant enzymes including catalase and superoxide dismutase, which directly scavenge reactive oxygen species (Cheng et al., 2024; Zhang et al., 2016; Kodali et al., 2024). The antioxidant effects are further enhanced through the delivery of siRNA targeting early growth response-1, which modulates oxidative stress levels and promotes mitophagy regulation (Huang et al., 2025).

HUCMSCs-Exos also regulate ferroptosis, an iron-dependent form of programmed cell death characterized by lipid peroxidation. Exosomal miR-23a-3p inhibits ferroptosis by targeting divalent metal transporter 1, thereby reducing iron accumulation and subsequent oxidative damage (Song et al., 2021). Additionally, these exosomes protect against endoplasmic reticulum stress-induced apoptosis through activation of the phosphatidylinositol-3-kinase/AKT pathway, demonstrating their capacity to maintain cellular homeostasis under stress conditions (Zhang C. et al., 2020).

### 4.6 Promotion of cardiomyocyte proliferation and regeneration

The limited regenerative capacity of adult cardiomyocytes represents a fundamental challenge in cardiac repair, and emerging evidence suggests that HUCMSCs-Exos can promote cardiomyocyte proliferation through sophisticated mechanisms. The coordination of cell-cycle reentry and ribosome biogenesis represents a critical network for cardiac repair, with exosomal circASXL1 playing a pivotal role in regulating both cyclin-dependent kinases 6/retinoblastoma-mediated cell-cycle reentry and nucleolin/ribosome biogenesis-mediated cytokinesis (Wang Y. et al., 2024). These mechanisms synergistically enable the formation of functional mononucleated cardiomyocytes rather than dysfunctional binucleated cells, representing a significant advancement in regenerative cardiology. Moreover, the delivery of specific circular RNAs, such as circ-0001273, further enhances cardiomyocyte survival and proliferation in ischemic environments (Li et al., 2020). These findings suggest that HUCMSCs-Exos may overcome the traditional limitations of adult cardiac regeneration.

While exosome-mediated cardiomyocyte proliferation represents a significant advance, important limitations exist regarding cell-cycle re-entry in adult human myocardium. Human adult cardiomyocytes exhibit profound cell-cycle arrest with less than 1% annual turnover, compared to higher rates in rodent models (Bergmann et al., 2015). The polyploid and multinucleated nature of adult human cardiomyocytes presents additional barriers, as cell-cycle re-entry often results in nuclear division without cytokinesis (Leone and Engel, 2019). Furthermore, forced cell-cycle re-entry carries theoretical risks of dedifferentiation and arrhythmogenesis, as proliferating cardiomyocytes temporarily lose contractile function (Mohamed et al., 2018).

### 4.7 Enhancement of aged cell function and rejuvenation

An innovative aspect of HUCMSCs-Exos therapy involves their capacity to rejuvenate aged mesenchymal stem cells and enhance their therapeutic potential. HUCMSCs-Exos can transfer miR-136 to aged bone marrow MSCs, effectively reducing senescence markers including p53, p21, and p16 while enhancing proliferation, migration, and differentiation capabilities. This rejuvenation effect is mediated through the downregulation of apoptotic peptidase activating factor, a downstream target of miR-136 (Zhang N. et al., 2020).

This mechanism is particularly relevant for clinical applications, as it suggests that HUCMSCs-Exos could enhance the therapeutic efficacy of autologous cell therapies in elderly patients, where cellular senescence typically limits treatment effectiveness. The ability to restore youthful characteristics to aged cells represents a paradigm shift in regenerative medicine approaches. While these preclinical findings demonstrate substantial therapeutic potential, successful clinical translation requires addressing several key challenges.

## 5 Clinical translation: opportunities and challenges

The translation of HUCMSCs-Exos from promising preclinical findings to clinical applications presents both unprecedented opportunities and significant challenges that must be systematically addressed to realize their therapeutic potential.

### 5.1 Current clinical translation status

The clinical translation of HUCMSCs-Exos for MI treatment is currently in its early phases, with several factors contributing to this gradual progression from bench to bedside. Safety evaluation studies have demonstrated that HUCMSCs-Exos are well-tolerated in animal models, showing no adverse effects on liver or renal function, hemolysis, vascular stimulation, or systemic anaphylaxis (Sun et al., 2016). These comprehensive safety profiles provide a solid foundation for clinical trial initiation.

The therapeutic mechanisms demonstrated in preclinical studies encompass multiple cardioprotective pathways, suggesting broad clinical applicability. However, the complexity of these mechanisms also presents challenges in identifying optimal patient populations and treatment protocols. Current research focuses on establishing standardized protocols for exosome isolation, characterization, and administration that can be reliably reproduced across different clinical centers.

### 5.2 Manufacturing and standardization challenges

One of the primary obstacles in clinical translation involves the standardization of HUCMSCs-Exos production and characterization. While ultracentrifugation remains the most commonly used isolation technique, concerns regarding non-vesicular contamination have led to the exploration of alternative methods such as chromatography, which demonstrates reduced contamination levels. The development of scalable production methods, including asymmetric flow field-flow fractionation, offers promising alternatives for clinical-grade manufacturing (Gardiner et al., 2016; Yang et al., 2019).

Quality control poses another critical challenge, requiring comprehensive characterization protocols that assess physical parameters, biochemical markers, and functional activities. The establishment of standardized good manufacturing practice protocols for HUCMSCs-Exos production is essential for regulatory approval and clinical implementation. Additionally, the development of potency assays that accurately predict therapeutic efficacy remains an active area of research.

Notably, manufacturing standardization according to Good Manufacturing Practice requirements presents complex challenges. The International Society for Extracellular Vesicles 2023 guidelines mandate comprehensive characterization including particle concentration, size distribution, surface marker expression (CD63, CD81, CD9), and purity assessment (Welsh et al., 2024). Current GMP-compliant production achieves yields of approximately 1 × 10^11^ particles per run, with tangential flow filtration demonstrating superior scalability (Yang et al., 2019; Buschmann et al., 2021). However, batch release criteria remain inconsistent across facilities (Gardiner et al., 2016). A number of clinical trials examining the therapeutic potential of HUCMSCs in cardiovascular diseases have been registered to date. These include studies evaluating the use of HUCMSCs for the treatment of heart failure (NCT04939077), the safety and efficacy of intravenous HUCMSCs infusion in patients with heart failure and reduced ejection fraction (NCT04992832), and the combined application of allogeneic HUCMSCs with injectable collagen scaffolds in patients with chronic ischemic cardiomyopathy (NCT02635464) (https://clinicaltrials.gov). While no clinical investigation has directly assessed the role of HUCMSCs-Exos in MI, the findings from these trials offer a valuable theoretical foundation that supports the future development of targeted and precise therapeutic strategies involving HUCMSCs-Exos for MI treatment.

### 5.3 Delivery and retention optimization

The short half-life and rapid clearance of exosomes following administration pose significant challenges for clinical efficacy. Innovative delivery strategies have been developed to address these limitations, including the use of biomaterial-based carriers that enhance retention and stability. Peptide hydrogel encapsulation has demonstrated the ability to provide sustained and controlled release of HUCMSCs-Exos while improving myocardial function through reduced inflammation, fibrosis, and apoptosis (Han et al., 2019).

Microneedle patch systems offer another promising delivery approach, providing localized administration with improved retention in infarcted myocardium (Yuan et al., 2023; Huang et al., 2025). Conductive hydrogels that anchor exosomes through specific peptide interactions have shown superior therapeutic effects by prolonging exosome retention while matching the electrical properties of native myocardium (Zou et al., 2021). These delivery innovations represent crucial advances toward clinical implementation.

### 5.4 Regulatory pathway and clinical trial design

The regulatory pathway for HUCMSCs-Exos involves unique considerations due to their classification as biological products with complex compositions. Regulatory agencies require comprehensive data on manufacturing processes, quality control measures, and safety profiles before approving clinical trials. The heterogeneous nature of exosome populations necessitates sophisticated analytical methods for characterization and consistency assessment.

Clinical trial design must consider the multifaceted mechanisms of action and potential patient variability in response. Appropriate endpoints must be selected that capture both short-term safety and long-term efficacy outcomes. The timing of administration, dosing regimens, and combination therapies require careful optimization based on preclinical findings and early-phase clinical data.

### 5.5 Cost-effectiveness and healthcare integration

The economic considerations of HUCMSCs-Exos therapy include both production costs and potential healthcare savings from improved patient outcomes. While initial production costs may be substantial, the potential for reduced hospitalization, decreased need for advanced heart failure therapies, and improved quality of life could provide significant economic benefits. Cost-effectiveness analyses will be essential for healthcare system adoption and reimbursement decisions.

Integration into existing clinical workflows requires consideration of logistical factors including storage, handling, and administration protocols. The development of point-of-care testing methods for exosome quality assessment could facilitate clinical implementation while ensuring therapeutic consistency.

### 5.6 Current limitations and unresolved challenges

Despite promising preclinical evidence, several critical limitations must be addressed before widespread clinical implementation. Batch-to-batch variability remains significant, with studies reporting 20%–30% variation in particle concentration and cargo content between production lots (Gardiner et al., 2016; Welsh et al., 2024). Long-term safety data beyond 12 months are notably absent, raising concerns about potential immunological responses and biodistribution patterns (Sun et al., 2016). The optimal therapeutic window and dosing regimen remain undefined, with current protocols based primarily on preclinical extrapolations (Willis et al., 2017). Manufacturing costs using current GMP protocols are estimated at $10,000–20,000 per dose, potentially limiting accessibility (Aijaz et al., 2018). Additionally, emerging evidence from stem cell-derived extracellular vesicles in wound healing highlights potential systemic effects requiring monitoring, as exosome biodistribution can extend beyond target tissues (Ha et al., 2020).

Furthermore, the immunomodulatory effects extend beyond macrophage polarization to encompass dendritic cell regulation, critical for bridging innate and adaptive immunity in post-MI healing. Dendritic cells (DCs) accumulate in infarcted myocardium within 24–48 h, processing cardiac antigens and potentially triggering autoimmune responses (Wang et al., 2022). HUCMSCs-Exos modulate DCs maturation through delivery of immunosuppressive factors including TGF-β1 and IL-10 (Zhang et al., 2016; Kodali et al., 2024; Bi et al., 2022; Raghav and Mann, 2021), shifting DCs toward tolerogenic phenotypes that promote regulatory T cell expansion (Wang et al., 2022). This parallels mechanisms in extracellular vesicle-based wound healing, where coordinated immune regulation enables regeneration (Ha et al., 2020; Mann et al., 2022). Additionally, exosomal miRNAs can target cancer stem cells through shared signaling pathways, highlighting the need for careful evaluation of off-target effects (Shen et al., 2019; Raghav and Mann, 2024).

## 6 Engineered exosomes: the next-generation therapeutics

The evolution of exosome-based therapeutics has progressed from naturally secreted vesicles to sophisticated engineered platforms that enhance therapeutic efficacy through targeted modifications and optimized delivery systems.

### 6.1 Genetic engineering approaches

Advanced genetic engineering strategies have revolutionized the therapeutic potential of HUCMSCs-Exos by enabling precise modification of their cargo and functional properties. Overexpression of specific factors in parent cells provides a direct method for enhancing exosome therapeutic content. At 28 days following MI, MIF-engineered HUCMSCs-derived exosomes significantly enhanced capillary density by twofold and reduced infarct size by threefold through the upregulation of miR-133a-3p, as compared to natural exosomes (Zhu et al., 2021).

TIMP2 overexpression in HUCMSCs produces exosomes with enhanced anti-remodeling properties through the AKT/Sfrp2 pathway, demonstrating enhanced therapeutic potential in restricting extracellular matrix remodeling and improving cardiac function. These engineered exosomes exhibit a statistically significant approximately 30% increase in angiogenic potential and a comparable approximately 40% decrease in apoptotic activity when compared to native exosomes, accompanied by additional cardioprotective benefits (Ni et al., 2019).

AKT modification represents another successful engineering approach, with AKT-overexpressing HUCMSCs producing exosomes with enhanced angiogenic capacity through PDGF-D upregulation. Quantitative analysis further reveals that the extent of new blood vessel formation in this condition is approximately twice that observed in the presence of natural exosomes (Ma et al., 2017). These examples demonstrate the potential for targeted genetic modifications to enhance specific therapeutic properties.

### 6.2 MiRNA engineering and cargo optimization

MiRNA engineering represents a particularly promising approach for enhancing HUCMSCs-Exos therapeutic efficacy. Transfection of specific miRNA mimics or inhibitors into parent cells enables precise modulation of exosome cargo composition. miR-133a-3p engineering has demonstrated enhanced myocardial repair effects through improved angiogenesis and reduced apoptosis (Sun et al., 2024). Similarly, miR-29b loading via electroporation provides potent anti-fibrotic activity for preventing excessive cardiac fibrosis (Yuan et al., 2023).

The therapeutic potential extends to complex regulatory networks, with engineered exosomes carrying specific circular RNAs such as circ-0001273 demonstrating enhanced anti-apoptotic effects (Li et al., 2020). Long non-coding RNAs, including lncRNA UCA1, can be enhanced in exosomes to provide superior cytoprotective effects through competitive endogenous RNA mechanisms (Diao and Zhang, 2021). These approaches demonstrate the sophisticated cargo engineering possibilities for optimizing therapeutic outcomes.

### 6.3 Surface engineering and targeting strategies

Surface engineering of exosomes enables enhanced targeting specificity and cellular uptake efficiency. Cardiac-targeting peptides can be covalently attached to exosome surfaces, creating biomimetic nanovesicles with enhanced myocardial targeting capability (Zhang J. et al., 2024). These engineered vesicles demonstrate superior accumulation in ischemic hearts and improved therapeutic efficacy compared to unmodified exosomes.

The development of multi-functional surface modifications that combine targeting capabilities with therapeutic cargo represents an advanced engineering approach. Integration of placental growth factors with cardiac-targeting peptides creates comprehensive therapeutic platforms that address multiple aspects of cardiac repair simultaneously (Zhang J. et al., 2024). These sophisticated engineering approaches demonstrate the potential for creating highly specialized therapeutic vehicles.

### 6.4 Biomaterial integration and sustained release systems

The integration of engineered exosomes with advanced biomaterial systems represents a major advancement in therapeutic delivery. Peptide hydrogel systems provide sustained release capabilities while protecting exosome integrity and enhancing therapeutic duration. The PA-GHRPS/NapFF peptide combination creates hydrogels with optimal gelation properties for exosome encapsulation and controlled release (Han et al., 2019).

Microneedle patch systems offer minimally invasive delivery with enhanced local retention. Gelatin-based microneedles loaded with engineered exosomes demonstrate superior therapeutic outcomes through sustained local delivery and reduced systemic clearance (Yuan et al., 2023; Huang et al., 2025). Conductive hydrogel systems that incorporate aniline tetramer modifications provide electrical conductivity matching native myocardium while serving as exosome anchoring platforms (Zou et al., 2021).

### 6.5 Multi-component therapeutic systems

Advanced engineering approaches involve the creation of multi-component systems that combine multiple therapeutic modalities. The integration of TGF-β3 with HUCMSCs-Exos in nanofibrous cardiac patches demonstrates enhanced cardiac regeneration through synergistic effects on angiogenesis and mesenchymal differentiation (Ping et al., 2023). These composite systems address multiple aspects of cardiac repair simultaneously.

Normothermic *ex vivo* heart perfusion systems enhanced with HUCMSCs-Exos represent sophisticated applications for organ preservation and transplantation. These systems demonstrate improved graft function through enhanced cardioprotection and reduced ischemia-reperfusion injury (Zhang Z. et al., 2024). Such applications highlight the versatility of engineered exosome systems for diverse clinical scenarios. The results of preclinical studies on HUCMSCs-Exos in MI is summarized in Table 1.

**TABLE 1:** Summary of preclinical studies on HUCMSCs-Exos in MI.

Therapeutic Cargo/Engineering approach	Molecular mechanisms	Primary outcomes	Route of administration	Animal models	Major findings	References
Native exosomes	Bcl-2 family protein regulation	Apoptosis inhibition, angiogenesis enhancement	Intravenous injection	Rat AMI model	Improved cardiac contractility with reduced fibrosis	Zhao et al. (2015)
miR-19a	SOX6/AKT-JNK3/caspase-3 modulation	Apoptosis inhibition	Intravenous injection	Rat AMI model	AKT pathway activation through SOX6 targeting	Huang L. et al. (2020)
miR-125b-5p	Smad7 upregulation	Myocardial repair promotion	Intravenous injection	Rat AMI model	Enhanced Smad7 expression through miR-125b-5p modulation	Wang et al. (2018)
lncRNA UCA1	miR-143/Bcl-2/Beclin-1 axis	Hypoxia/reoxygenation injury protection	Intravenous injection	Rat I/R model	Bcl-2 upregulation through competitive miR-143 binding	Diao and Zhang (2021)
MIF overexpression + miR-133a-3p	AKT signaling pathway activation	Angiogenesis promotion, apoptosis inhibition	Intravenous injection	Rat AMI model	Significant improvement in cardiac function and neovascularization	Zhu et al. (2021)
Hypoxic preconditioning + miR-214	Sufu/Hedgehog pathway activation	Angiogenesis enhancement	Intramyocardial injection	Rat MI model	Improved angiogenic capacity through hypoxic conditioning	Shao et al. (2023)
miR-1246	PRSS23/Snail-α-SMA pathway modulation	Angiogenesis enhancement	Intravenous injection	Rat CHF model	Increased myocardial vascularization with heart failure improvement	Wang et al. (2021)
AKT overexpression	PDGF-D upregulation	Cardiac regeneration, angiogenesis promotion	Intravenous injection	Rat LAD ligation model	Enhanced endothelial cell function and proliferation	Ma et al. (2017)
miR-24-3p	Plcb3/NF-κB pathway inhibition	M2 macrophage polarization	Intramyocardial injection	Mouse MI model	Reduced excessive inflammation with enhanced cardiac repair	Zhu et al. (2022)
miR-204	Macrophage polarization modulation	M1 to M2 phenotype switching	Intravenous injection	Mouse I/R model	Suppressed M1 with enhanced M2 polarization	Yuan et al. (2022)
Native exosomes	NLRP3 inflammasome/caspase-1 inhibition	Pyroptosis suppression	Intravenous injection	Rat I/R model	Anti-inflammatory effects with significant reduction of pyroptosis markers	Diao et al. (2024)
miR-100-5p	FOXO3/NLRP3 pathway inhibition	Pyroptosis protection	*In vitro* co-culture	AC16 cell H/R model	Protection against pyroptotic cardiomyocyte injury	Liang et al. (2021)
miR-29b	Anti-fibrotic protein downregulation	Prevention of cardiac fibrosis	Gelatin microneedle patch	Mouse MI model	Enhanced exosome retention with significant fibrosis reduction	Yuan et al. (2023)
Native exosomes	Fibroblast differentiation regulation	Anti-inflammatory phenotype promotion	Intramyocardial injection	Rat MI model	Enhanced fibroblast-to-myofibroblast transition	Shi et al. (2019)
TIMP2 overexpression	AKT/Sfrp2 pathway activation	ECM remodeling inhibition, antioxidant effects	Intravenous injection	Rat MI model	Restricted ECM remodeling with increased SOD and GSH levels	Ni et al. (2019)
Modified exosomes (IMTP-EXO)	PI3K/AKT pathway activation	Reversal of ventricular remodeling	Intravenous injection	Rat I/R model	Sustained improvement in EF and FS with reduced LVEDD	Wang et al. (2025)
siEGR1	Oxidative stress modulation, mitophagy enhancement	Antioxidant effects, mitophagy promotion	GelMA microneedle patch	Mouse I/R model	Significant cardiac function improvement with reduced fibrosis	Huang et al. (2025)
miR-23a-3p	DMT1 inhibition	Ferroptosis suppression	Intravenous injection	Mouse AMI model	Reduced iron accumulation and lipid peroxidation	Song et al. (2021)
Native exosomes	PI3K/AKT pathway activation	ER stress alleviation	*In vitro* treatment	H9c2 cell H/R model	Reduced ER stress-induced apoptosis	Zhang C. et al. (2020)
circASXL1	CDK6/Rb1 and Ncl/ribosome biogenesis	Cell cycle re-entry, cytokinesis promotion	Intramyocardial injection	Mouse MI model	Enhanced cardiomyocyte proliferation and division	Wang Y. et al. (2024)
circ-0001273	Anti-apoptotic pathway activation	Cardiomyocyte survival enhancement	Intramyocardial injection	Rat MI model	Accelerated MI repair through circular RNA delivery	Li et al. (2020)
miR-136	Apaf1 downregulation	Cellular rejuvenation, senescence reversal	Intramyocardial injection	Mouse MI model	Reversal of MSC aging phenotype	Zhang N. et al. (2020)
Native exosomes	Safety evaluation	No adverse effects observed	Intravenous injection	Rabbit, guinea pig, rat models	Absence of hemolysis, vascular irritation, or allergic reactions	Sun et al. (2016)
Native exosomes	Multiple cardioprotective pathways	Reduction of inflammation, fibrosis, and apoptosis	PA-GHRPS peptide hydrogel	Rat MI model	Sustained exosome release with prolonged therapeutic effects	Han et al. (2019)
CP05 peptide anchoring	Multiple cardiac repair mechanisms	Cardiac function improvement, fibrosis reduction	Conductive hydrogel platform	Rat MI model	Enhanced exosome retention with matched myocardial conductivity	Zou et al. (2021)
miR-133a-3p engineering	Anti-apoptotic and pro-angiogenic pathways	Myocardial repair enhancement	Peri-infarct injection	Rat AMI model	Significant LVEF increase with fibrosis reduction	Sun et al. (2024)
PLGF + CHP peptide	Cardiac-targeted delivery	Myocardial survival, neovascularization	Biomimetic nanovesicles	Mouse MI model	Improved therapeutic specificity through cardiac targeting	Zhang J. et al. (2024)
TGF-β3 co-delivery	Angiogenesis and mesenchymal differentiation	Cardiac regeneration enhancement	PCL/COL-1 nanofibrous patch	Rat AMI model	Significant increase in left ventricular ejection fraction	Ping et al. (2023)
Native exosomes	PI3K/AKT pathway activation	Transplant cardioprotection	Normothermic *ex* *vivo* perfusion	Rat cardiac transplant model	Improved DCD heart graft function	Zhang Z. et al. (2024)

Abbreviations: ADMSCs, adipose-derived mesenchymal stem cells; AKT, protein kinase B; AMI, acute myocardial infarction; Apaf1, apoptotic peptidase activating factor 1; bFGF, basic fibroblast growth factor; CDK6, cyclin-dependent kinase 6; CHF, chronic heart failure; CHP, cardiac homing peptide; CMEC, cardiac microvascular endothelial cells; COL-1, type I collagen; DCD, donation after circulatory death; DMT1, divalent metal transporter 1; ECM, extracellular matrix; EF, ejection fraction; EGR1, early growth response protein 1; ER, endoplasmic reticulum; FS, fractional shortening; GelMA, gelatin methacryloyl; GSH, glutathione; H/R, hypoxia/reoxygenation; HGF, hepatocyte growth factor; HUCMSCs-Exos, human umbilical cord mesenchymal stem cells-derived exosomes; I/R, ischemia/reperfusion; LAD, left anterior descending coronary artery; lncRNA, long non-coding RNA; LVEDD, left ventricular end-diastolic dimension; LVEF, left ventricular ejection fraction; MI, myocardial infarction; MIF, macrophage migration inhibitory factor; miR, microRNA; ncl, nucleolin; NF-κB, nuclear factor kappa B; NLRP3, NLR, family pyrin domain containing 3; PA-GHRPS, self-assembling peptide; PCL, polycaprolactone; PDGF-D, platelet-derived growth factor D; PI3K, phosphoinositide 3-kinase; Plcb3, phospholipase C beta 3; PLGF, placental growth factor; PRSS23, serine protease 23; Rb1, retinoblastoma protein 1; Sfrp2, secreted frizzled-related protein 2; SOD, superoxide dismutase; SOX6, SRY-box, transcription factor 6; Sufu, suppressor of fused; TGF-β3, transforming growth factor beta 3; TIMP2, tissue inhibitor of metalloproteinases 2; UCA1, urothelial cancer associated 1; VEGF, vascular endothelial growth factor; α-SMA, alpha-smooth muscle actin.

### 6.6 Advantages of engineered approaches

Engineered HUCMSCs-Exos offer several distinct advantages over naturally secreted vesicles. Enhanced targeting specificity reduces off-target effects while improving therapeutic efficacy at lower doses. Optimized cargo composition enables precise modulation of specific pathways relevant to cardiac repair. Sustained release formulations extend therapeutic duration while reducing administration frequency.

The ability to combine multiple therapeutic modalities within single delivery systems provides comprehensive treatment approaches that address the complex pathophysiology of MI. Quality control and reproducibility are enhanced through standardized engineering protocols, facilitating regulatory approval and clinical implementation. These advantages position engineered exosomes as the next-generation of cardiac therapeutics. These engineering advances, combined with emerging technologies, point toward exciting future directions for the field. The results of engineered HUCMSCs-Exos in MI is summarized in Table 2.

**TABLE 2:** Summary of engineered HUCMSCs-Exos in MI: Strategies, advantages, and therapeutic outcomes.

Engineering Strategy	Specific Modification/Method	Key advantages	Major therapeutic outcomes	Delivery System/Carrier	References
Genetic engineering					
MIF overexpression	Enhanced miR-133a-3p content	Superior angiogenic capacity; Enhanced anti-apoptotic effects	Improved cardiac function with significant neovascularization	Intravenous injection	Zhu et al. (2021)
AKT modification	PDGF-D upregulation	Enhanced angiogenic capacity; Improved endothelial cell function	Superior endothelial cell proliferation, migration, and tube formation	Intravenous injection	Ma et al. (2017)
TIMP2 overexpression	AKT/Sfrp2 pathway activation	Enhanced anti-remodeling properties; Restricted ECM degradation	Superior efficacy in preventing adverse cardiac remodeling	Intravenous injection	Ni et al. (2019)
RNA Engineering					
lncRNA UCA1 enhancement	Competitive endogenous RNA mechanism	Superior cytoprotective effects; miR-143 competitive binding	Enhanced Bcl-2 expression and cell survival	Intravenous injection	Diao and Zhang (2021)
miR-29b loading	Electroporation-mediated loading	Potent anti-fibrotic activity; Enhanced retention	Prevention of excessive cardiac fibrosis	Gelatin microneedle patch	Yuan et al. (2023)
siRNA delivery	siEGR1 incorporation	Oxidative stress modulation; Enhanced mitophagy	Improved cardiac function with reduced fibrosis	GelMA microneedle patch	Huang et al. (2025)
circRNA engineering	circ-0001273 delivery	Enhanced anti-apoptotic effects; Improved cardiomyocyte survival	Accelerated MI repair through circular RNA mechanisms	Intramyocardial injection	Li et al. (2020)
miR-133a-3p engineering	Direct miRNA loading	Enhanced myocardial repair; Improved targeting specificity	Significant LVEF increase with reduced fibrosis	Peri-infarct injection	Sun et al. (2024)
Biomaterial Integration					
Microneedle patches	Gelatin-based microneedles	Minimally invasive delivery; Enhanced local retention	Superior therapeutic outcomes through sustained local delivery	Transdermal patch	Yuan et al. (2023), Huang et al. (2025)
Peptide hydrogel encapsulation	PA-GHRPS/NapFF combination	Sustained release; Protected exosome integrity	Prolonged therapeutic duration with reduced inflammation	Injectable hydrogel	Han et al. (2019)
Conductive hydrogels	Aniline tetramer modification	Electrical conductivity matching myocardium; Exosome anchoring	Enhanced cardiac function with reduced fibrosis	Injectable conductive gel	Zou et al. (2021)
Multi-Component Systems					
Growth factor co-delivery	TGF-β3 integration	Synergistic effects on angiogenesis; Enhanced mesenchymal differentiation	Significant increase in LVEF and cardiac regeneration	PCL/COL-1 nanofibrous patch	Ping et al. (2023)
*Ex vivo* perfusion enhancement	Normothermic heart perfusion with exosomes	Improved graft preservation; Reduced I/R injury	Enhanced DCD heart graft function and viability	Organ perfusion system	Zhang et al. (2024b)
Advantages Over Native Exosomes					
Quality control and reproducibility	Standardized engineering protocols	Consistent therapeutic potency; Facilitated regulatory approval	Reliable clinical translation potential	All engineered systems	Gardiner et al. (2016), Yang et al. (2019), Welsh et al. (2024)
Optimized cargo composition	Precise pathway modulation	Targeted therapeutic effects; Predictable outcomes	Superior efficacy in specific pathways	Various approaches	Zhu et al. (2021), Ma et al. (2017), Ni et al. (2019)
Sustained release formulations	Biomaterial integration	Extended therapeutic duration; Reduced administration frequency	Long-term cardioprotective effects	Hydrogels, patches	Han et al. (2019), Zou et al. (2021)
Enhanced targeting specificity	Various targeting modifications	Reduced off-target effects; Lower effective doses required	Improved therapeutic index and safety profile	Multiple systems	Zhang et al. (2024a)

Abbreviations: AKT, protein kinase B; CHP, cardiac homing peptide; COL-1, type I collagen; CP05, cardiac-targeting peptide 05; DCD, donation after circulatory death; ECM, extracellular matrix; EGR1, early growth response protein 1; GelMA, gelatin methacryloyl; HUCMSCs-Exos, human umbilical cord mesenchymal stem cells-derived exosomes; I/R, ischemia/reperfusion; lncRNA, long non-coding RNA; LVEF, left ventricular ejection fraction; MI, myocardial infarction; MIF, macrophage migration inhibitory factor; miR, microRNA; PA-GHRPS, self-assembling peptide; PCL, polycaprolactone; PDGF-D, platelet-derived growth factor D; PLGF, placental growth factor; Sfrp2, secreted frizzled-related protein 2; siRNA, small interfering RNA; TGF-β3, transforming growth factor beta 3; TIMP2, tissue inhibitor of metalloproteinases 2; UCA1, urothelial cancer associated 1.

However, critical distinction must be made between preclinical innovations and clinically validated approaches. Currently, all engineering strategies described remain in preclinical development stages, with no engineered exosome formulations advancing beyond large animal studies. Surface functionalization and genetic modification demonstrate proof-of-concept in rodent models but face substantial regulatory hurdles regarding safety validation and manufacturing consistency. Only native, unmodified exosomes approach clinical readiness, with engineered variants requiring additional years of development before potential clinical trials. This timeline reflects necessary safety studies, manufacturing scale-up, and regulatory negotiations for these complex biological products.

## 7 Future directions and perspectives

The advancement of HUCMSCs-Exos therapy requires focused efforts in three priority areas that address current translational barriers. First, establishing standardized characterization and potency assays aligned with regulatory requirements represents the most immediate need, requiring collaborative development of reference materials and validated analytical methods. Second, optimization of manufacturing processes to achieve cost-effective scale-up while maintaining therapeutic quality necessitates innovation in bioreactor design and downstream processing technologies. Third, design of appropriately powered clinical trials with mechanistically informed endpoints must bridge the gap between preclinical promise and clinical validation, requiring careful patient stratification and biomarker-guided treatment protocols. These priorities should guide resource allocation and research efforts over the next 5–10 years to enable successful clinical translation.

### 7.1 Advanced mechanistic understanding

Future research should focus on elucidating the complete molecular networks underlying HUCMSCs-Exos therapeutic effects. While current studies have identified numerous individual pathways, comprehensive systems biology approaches are needed to understand the integrated responses and potential synergistic interactions. Single-cell RNA sequencing of target cells following exosome treatment could provide unprecedented insights into cellular response heterogeneity and identify novel therapeutic targets.

The development of real-time imaging techniques for tracking exosome distribution, uptake, and cargo release *in vivo* represents a critical research priority. Advanced imaging modalities, including photoacoustic imaging and magnetic resonance imaging with exosome-specific contrast agents, could provide valuable insights into therapeutic kinetics and optimization strategies. Understanding the temporal dynamics of therapeutic effects will enable optimized timing and dosing protocols.

### 7.2 Personalized medicine approaches

The heterogeneity of MI presentations and patient characteristics requires personalized therapeutic approaches. Future research should focus on identifying patient-specific factors that influence HUCMSCs-Exos therapeutic efficacy, including genetic polymorphisms, comorbidities, and baseline cardiac function. The development of companion diagnostics that predict treatment response could enable precision medicine applications.

Autologous versus allogeneic exosome therapy represents an important consideration for personalized approaches. While allogeneic HUCMSCs-Exos offer advantages in terms of standardization and immediate availability, autologous approaches may provide superior compatibility and efficacy in certain patient populations. The rejuvenation effects of HUCMSCs-Exos on aged cells suggest potential for enhancing autologous therapies in elderly patients (Zhang N. et al., 2020).

### 7.3 Combination therapeutic strategies

The integration of HUCMSCs-Exos with established cardiac therapies represents a promising direction for enhanced therapeutic outcomes. Combination with pharmacological agents, including angiotensin converting enzyme inhibitors, beta-blockers, and anti-platelet therapies, could provide synergistic cardioprotective effects. The timing and sequencing of combination therapies require careful optimization to maximize benefits while minimizing potential interactions.

Cell therapy combinations offer another exciting opportunity, with HUCMSCs-Exos potentially serving as priming agents that enhance the therapeutic efficacy of subsequent cell transplantation. The demonstrated ability of exosomes to rejuvenate aged cells suggests applications for enhancing autologous cell therapy outcomes (Zhang N. et al., 2020). Tissue engineering approaches that incorporate HUCMSCs-Exos into cardiac patches or scaffolds could provide sustained therapeutic delivery with enhanced integration (Yuan et al., 2023; Ping et al., 2023).

### 7.4 Advanced engineering and manufacturing

Future developments in exosome engineering should focus on creating increasingly sophisticated therapeutic platforms. CRISPR-Cas9 technology could enable precise genetic modifications for enhanced therapeutic cargo production. Synthetic biology approaches might allow for the creation of designer exosomes with optimized properties for specific therapeutic applications.

**FIGURE 3:**
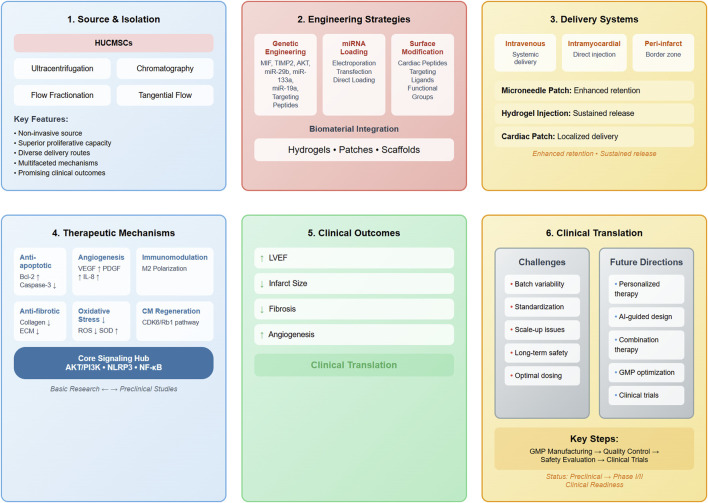
Comprehensive translational roadmap of HUCMSCs-Exos from bench to bedside in MI treatment. This integrated schematic diagram systematically presents the complete translational pathway of HUCMSCs-Exos therapy through six interconnected modules: (1) Source & Isolation, depicting HUCMSCs as the cell source and various isolation techniques; (2) Engineering Strategies, illustrating genetic engineering, miRNA loading, surface modification, and biomaterial integration approaches; (3) Delivery Systems, showing different administration routes and advanced delivery vehicles; (4) Therapeutic Mechanisms, highlighting six primary cardioprotective pathways converging on three core signaling hubs (AKT/PI3K, NLRP3, and NF-κB); (5) Clinical Outcomes, summarizing key therapeutic efficacy parameters; and (6) Clinical Translation, presenting current challenges and future directions with the progression pipeline from GMP manufacturing to clinical trials. The diagram emphasizes the systematic transition from basic research through preclinical studies to Phase I/II clinical readiness. Abbreviations: AI, artificial intelligence; AKT, protein kinase B; Bcl-2, B-cell lymphoma 2; CDK6, cyclin-dependent kinase 6; CM, cardiomyocyte; ECM, extracellular matrix; GMP, Good Manufacturing Practice; IL-8, interleukin-8; LVEF, left ventricular ejection fraction; MIF, macrophage migration inhibitory factor; miR, microRNA; NF-κB, nuclear factor kappa B; NLRP3, NLR family pyrin domain containing 3; PDGF, platelet-derived growth factor; PI3K, phosphoinositide 3-kinase; Rb1, retinoblastoma protein 1; ROS, reactive oxygen species; SOD, superoxide dismutase; TIMP2, tissue inhibitor of metalloproteinases 2; VEGF, vascular endothelial growth factor.

Manufacturing advances should focus on developing cost-effective, scalable production methods that maintain therapeutic quality and consistency. Continuous manufacturing processes, automated quality control systems, and standardized characterization protocols will be essential for clinical implementation. The development of lyophilization and other preservation methods could enable long-term storage and global distribution (Gardiner et al., 2016; Yang et al., 2019; Welsh et al., 2024). Figure 3 delineates an integrated translational roadmap for HUCMSCs-Exos therapeutic development, systematically illustrating the multifaceted progression from bench-side mechanistic investigations through preclinical optimization to bedside clinical implementation in MI treatment.

## 8 Conclusion

### 8.1 Summary of key findings

This comprehensive review has systematically evaluated the current state of knowledge regarding HUCMSCs-Exos as a therapeutic intervention for MI, revealing a rapidly evolving field with substantial promise for clinical translation. The evidence demonstrates that HUCMSCs-Exos exert their cardioprotective effects through multiple interconnected mechanisms, including anti-apoptotic signaling, angiogenesis promotion, inflammatory modulation, anti-fibrotic activity, oxidative stress reduction, and enhancement of cardiac regeneration (Zhao et al., 2015; Zhu et al., 2021; Zhu et al., 2022; Diao et al., 2024; Yuan et al., 2023).

The mechanistic diversity of HUCMSCs-Exos therapeutic effects is particularly noteworthy, encompassing regulation of miRNAs (miR-29b, miR-133a-3p, miR-24-3p, miR-19a, miR-1246, miR-100-5p, miR-23a-3p, miR-204, miR-214), long non-coding RNAs (lncRNA UCA1), circular RNAs (circASXL1, circ-0001273), and protein factors (MIF, TIMP2, AKT, PDGF-D). This mechanistic complexity suggests that HUCMSCs-Exos function as comprehensive therapeutic platforms rather than single-target interventions, potentially explaining their enhanced therapeutic potential compared to conventional pharmacological approaches (Huang L. et al., 2020; Wang et al., 2018; Diao and Zhang, 2021; Wang et al., 2021; Zhu et al., 2022; Yuan et al., 2023; Wang Y. et al., 2024; Li et al., 2020; Zhang N. et al., 2020).

### 8.2 Clinical significance and therapeutic potential

The clinical significance of HUCMSCs-Exos extends beyond their demonstrated preclinical efficacy to encompass several unique advantages that position them as next-generation cardiac therapeutics. Their ability to cross biological barriers, reduced immunogenicity compared to cellular therapies, enhanced stability, and decreased risk of malignant transformation represent significant advantages over existing treatment modalities.

Particularly compelling is the demonstrated capacity of HUCMSCs-Exos to address multiple pathophysiological processes simultaneously, including the prevention of adverse cardiac remodeling, promotion of neovascularization, and enhancement of endogenous repair mechanisms. The long-term cardioprotective effects observed in preclinical studies, including reversal of ventricular remodeling and sustained improvement in cardiac function, suggest potential for disease-modifying rather than merely symptomatic treatment approaches.

### 8.3 Innovation in delivery and engineering

The advancement of engineered exosome technologies represents a paradigm shift toward precision medicine applications in cardiovascular therapeutics. The development of sophisticated delivery systems, including biomaterial-based carriers, microneedle patches, and conductive hydrogels, demonstrates the potential for optimized therapeutic delivery with enhanced retention and sustained release properties.

Genetic engineering approaches that enable precise modulation of exosome cargo composition provide unprecedented opportunities for therapeutic customization. The ability to enhance specific therapeutic properties through targeted modifications, combined with surface engineering for improved targeting specificity, positions engineered HUCMSCs-Exos as highly sophisticated therapeutic platforms capable of addressing diverse clinical scenarios.

### 8.4 Research limitations and critical considerations

Despite the substantial progress in understanding HUCMSCs-Exos therapeutic mechanisms, several limitations must be acknowledged. The majority of evidence is derived from preclinical studies, with limited clinical data available to confirm therapeutic efficacy and safety in human patients. The heterogeneity of experimental protocols across studies, including differences in isolation methods, characterization approaches, and administration routes, complicates direct comparisons and meta-analyses.

Standardization challenges remain significant obstacles to clinical translation, particularly regarding manufacturing processes, quality control measures, and potency assessments. The complex regulatory pathway for these novel therapeutics requires comprehensive safety and efficacy data that may extend development timelines. Long-term safety data, including potential immunological responses and effects on normal tissue function, require further investigation.

### 8.5 Future research priorities

Priority research areas should focus on advancing clinical translation through well-designed human studies that establish safety profiles and therapeutic efficacy. The development of standardized protocols for exosome production, characterization, and administration is essential for regulatory approval and clinical implementation. Mechanistic studies utilizing advanced technologies, including single-cell analyses and real-time imaging, will provide crucial insights for therapeutic optimization.

The integration of artificial intelligence and machine learning approaches could accelerate the identification of optimal therapeutic targets and personalized treatment strategies. Collaborative efforts between academic institutions, biotechnology companies, and regulatory agencies will be essential for overcoming the complex challenges associated with clinical translation.

### 8.6 Final perspective

HUCMSCs-Exos represent a transformative approach to MI treatment that addresses the fundamental limitations of current therapeutic options. Their unique combination of safety, efficacy, and mechanistic sophistication positions them as leading candidates for next-generation cardiac therapeutics. The convergence of advances in stem cell biology, nanotechnology, and precision medicine have created unprecedented opportunities for developing comprehensive treatment strategies that address the complex pathophysiology of MI. While challenges remain in the path to clinical implementation, the substantial body of preclinical evidence, combined with ongoing technological advances and regulatory science initiatives, provides a strong foundation for successful translation. The potential impact of HUCMSCs-Exos therapy extends beyond individual patient benefits to encompass significant implications for global cardiovascular health, particularly given the increasing prevalence of MI worldwide. The field has reached a pivotal point where continued research investment, collaborative efforts, and innovative approaches to clinical translation could yield transformative therapies that fundamentally alter the prognosis and quality of life for patients with MI. The promise of HUCMSCs-Exos as comprehensive cardiac therapeutics justifies sustained commitment to advancing this field toward clinical realization.

## Abbreviations

AKT/PKB, protein kinase B; Bcl-2, B cell lymphoma-2; DCs, dendritic cells; HUCMSCs, human umbilical cord mesenchymal stem cells; HUCMSCs-Exos, HUCMSCs-derived exosomes; IL-1β, interleukin-1β; MI, myocardial infarction; MIF, macrophage migration inhibitory factor; miRNAs, microRNAs; MSCs, mesenchymal stem cells; NLRP3, NLR family pyrin domain containing 3; PCI, percutaneous coronary intervention; PDGF-D, platelet-derived growth factor D; Sfrp2, secreted frizzled-related protein 2; TGF-β, transforming growth factor beta; TIMP2, tissue inhibitor of metalloproteinases 2.
